# Factors Affecting Oncological Outcomes in Upper Tract Urothelial Carcinoma Patients with Chronic Kidney Disease and End-Stage Renal Disease

**DOI:** 10.3390/biomedicines14030554

**Published:** 2026-02-28

**Authors:** Hung-Keng Li, Hsiang-Ying Lee, Hsin-Chih Yeh, Chao-Yuan Huang, Chung-Hsin Chen, Chao-Hsiang Chang, Chin-Chung Yeh, Han-Yu Weng, Ta-Yao Tai, Yao-Chou Tsai, Shu-Yu Wu, Yuan-Hong Jiang, Yu-Khun Lee, I-Hsuan Alan Chen, Jen-Tai Lin, Thomas Y. Hsueh, Bing-Juin Chiang, Yung-Tai Chen, Jen-Shu Tseng, Chia-Chang Wu, Ting-En Tai, Wei-Yu Lin, Shiu-Dong Chung

**Affiliations:** 1 Division of Urology, Department of Surgery, Far Eastern Memorial Hospital, New Taipei 220, Taiwan; purered119@gmail.com; 2 Department of Life Science, School of Life Science, College of Science, National Taiwan Normal University, Taipei 220, Taiwan; 3 Department of Urology, Kaohsiung Medical University Hospital, Kaohsiung 807, Taiwan; 4 Department of Urology, School of Medicine, College of Medicine, Kaohsiung Medical University, Kaohsiung 807, Taiwan; 5 Department of Urology, Kaohsiung Municipal Ta-Tung Hospital, Kaohsiung 807, Taiwan; 6 Graduate Institute of Clinical Medicine, College of Medicine, Kaohsiung Medical University, Kaohsiung 807,Taiwan; 7 Department of Urology, National Taiwan University Hospital, College of Medicine, National Taiwan University, Taipei 100, Taiwan; 8 Department of Urology, China Medical University and Hospital, Taichung 404, Taiwan; 9 School of Medicine, China Medical University, Taichung 404, Taiwan; 10 Department of Urology, National Cheng Kung University Hospital, College of Medicine, National Cheng Kung University, Tainan 701, Taiwan; 11 Department of Surgery, Taipei Tzu Chi Hospital, Buddhist Tzu Chi Medical Foundation, New Taipei 231, Taiwan; 12 Department of Urology, School of Medicine, Buddhist Tzu Chi University, Hualien 970, Taiwan; 13 Department of Urology, Taipei Medical University Hospital, Taipei Medical University, Taipei 110, Taiwan; 14 Department of Urology, Taipei Tzu Chi Hospital, Buddhist Tzu Chi Medical Foundation, New Taipei 231, Taiwan; 15 Department of Urology, Hualien Tzu Chi Hospital, Tzu Chi Medical Foundation, Tzu Chi University, Hualien 970, Taiwan; 16 Division of Urology, Department of Surgery, Kaohsiung Veterans General Hospital, Kaohsiung 813, Taiwan; 17 Division of Urology, Department of Surgery, Taipei City Hospital Renai Branch, Taipei 106, Taiwan; 18 Department of Urology, School of Medicine, National Yang Ming Chiao Tung University, Taipei 112, Taiwan; 19 College of Medicine, Fu-Jen Catholic University, New Taipei 242, Taiwan; 20 Department of Urology, Cardinal Tien Hospital, New Taipei 231, Taiwan; 21 Department of Urology, Postal Hospital, Taipei 100, Taiwan; 22 Department of Urology, Taiwan Adventist Hospital, Taipei 105, Taiwan; 23 Department of Urology, MacKay Memorial Hospital, Taipei 104, Taiwan; 24 Mackay Medical College, New Taipei 252, Taiwan; 25 Institute of Biomedical Informatics, National Yang Ming Chiao Tung University, Taipei 112, Taiwan; 26 Department of Urology, Shuang Ho Hospital, Taipei Medical University, New Taipei 235, Taiwan; 27 Department of Urology, School of Medicine, College of Medicine, Taipei Medical University, Taipei 110, Taiwan; 28 TMU Research Center of Urology and Kidney (TMU-RCUK), Taipei Medical University, Taipei 110,Taiwan; 29 Division of Urology, Department of Surgery, Chang Gung Memorial Hospital, Chiayi 613, Taiwan; 30 Chang Gung University of Science and Technology, Chiayi 613, Taiwan; 31 Department of Medicine, College of Medicine, Chang Gung University, Taoyuan 333, Taiwan; 32 Department of Nursing, College of Healthcare & Management, Asia Eastern University of Science and Technology, New Taipei 220, Taiwan; 33 General Education Center, Asia Eastern University of Science and Technology, New Taipei 220, Taiwan; 34 Graduate Institute of Medicine, Yuan Ze University, Taoyuan 320, Taiwan

**Keywords:** upper tract urothelial carcinoma, chronic kidney disease, end-stage renal disease

## Abstract

**Background/Objectives**: We assessed factors affecting the oncological outcomes in upper tract urothelial carcinoma patients with chronic kidney disease (CKD) and end-stage renal disease (ESRD) in Taiwan, using a large domestic upper tract urothelial carcinoma collaboration database. **Methods**: From July 1988 to December 2019, 15 hospitals joined the Taiwan Upper Tract Urothelial Carcinoma Collaboration Group. A total of 690 patients were included, and demographic, clinical, and pathological data were compared. Factors related to overall survival, cancer-specific survival, disease-free survival, and bladder recurrence-free survival were analyzed. **Results**: Out of the 690 patients, 605 had CKD and 85 had ESRD. In multivariate analysis, overall survival was associated with CKD stage (*p* = 0.024), age > 70 years (*p* = 0.002), and pathological stage III/IV (*p* = 0.014 and <0.001). Cancer-specific survival was associated with middle ureter tumors (*p* = 0.041), positive surgical margin (*p* = 0.005), and pathological stage III/IV (*p* = 0.010 and <0.001). Disease-free survival was associated with middle ureter tumors (*p* = 0.001), lower ureter tumors (*p* = 0.010), and pathological stage III/IV (*p* = 0.039 and <0.001). Female sex (*p* = 0.027), lower ureter tumors (*p* = 0.027), coronary artery disease (*p* = 0.047), and arrhythmias (*p* = 0.044) were associated with bladder recurrence-free survival. **Conclusions**: The oncological outcomes of UTUC patients with CKD and ESRD in Taiwan were affected by various factors. Tumor location and advanced pathological stage were related to OS, CSS, and DFS. Cardiac diseases were possibly related to BRFS.

## 1. Introduction

Urothelial carcinoma (UC) is one of the most common urological cancers, and upper tract UC (UTUC) accounts for 5~10% of all cases of UC in Western countries [1]. Interestingly, the incidence of UTUC is as high as 30~40% of all UC cases in Taiwan [2]. The prevalence of chronic renal disease (CKD) in Taiwan is also high at 11.9% [3]; CKD is a known significant risk factor for UTUC [4]. The aggressiveness of UTUC may increase in accordance with the severity of CKD [5]. Although the relationship between CKD and UTUC has been previously reported, most studies have been from single centers, and data from a larger source are lacking. To address the high prevalence of CKD and high incidence of UTUC in UC patients in Taiwan, a domestic study group including 15 hospitals, the Taiwan UTUC Collaboration Group, was founded with the goal of furthering research on the disease. Patients’ demographic data and treatment outcomes are recorded in the Taiwan UTUC Collaboration Group database. The aim of this study was to identify factors affecting the oncological outcomes of patients with UTUC, focusing on those with CKD and end-stage renal disease (ESRD), using data from this large multicenter database in Taiwan.

## 2. Materials and Methods

We retrospectively reviewed data of patients with UTUC from 15 participating Taiwanese Hospitals (Taipei Tzu Chi Hospital, Hualien Tzu Chi Hospital, Kaohsiung Medical University Hospital, Chang Gung Memorial Hospital, Chiayi, Kaohsiung Veterans General Hospital, China Medical University Hospital, Taipei City Hospital, National Taiwan University Hospital, Taipei Medical University–Shuang Ho Hospital, Taiwan Adventist Hospital, Cardinal Tien Hospital, Far Eastern Memorial Hospital, National Cheng Kung University Hospital, Taipei Medical University Hospital, and Mackay Memorial Hospital) in the Taiwan UTUC Collaboration Group between July 1988 and December 2019. We included patients with only upper tract tumors and excluded those with synchronous upper tract and bladder tumors. All individual identifiable information was removed. The study was conducted in accordance with the 1964 Declaration of Helsinki and strictly followed the guidelines of each institution.

A total of 2252 patients with UTUC were included in this study. Of these patients, 329 who did not undergo nephroureterectomy, 366 who were followed-up for <3 months, and 83 with missing estimated glomerular filtration rate (eGFR) data were excluded. We also excluded 514 patients with an eGFR > 60 mL/min/1.73 m^2^. Of the remaining 960 patients, 813 were assigned to the CKD group and 147 to the ESRD group. We further excluded 108 patients in the CKD group and 62 patients in the ESRD group who had synchronous UTUC and bladder tumors, and finally enrolled 605 patients in the CKD group and 85 patients in the ESRD group.

Patients’ renal function status was assessed at the time of UTUC diagnosis. CKD was defined as an eGFR between 60 mL/min/1.73 m^2^ and 15 mL/min/1.73 m^2^, and ESRD was defined as an eGFR < 15 mL/min/1.73 m^2^ using the Modification of Diet in Renal Disease (MDRD) equation. The demographic data included sex and age. Risk factors, medical disease comorbidities, initial presentation of the disease, tumor location, tumor size, tumor laterality, cell type, multiplicity, pre-operative cytology results, nephroureterectomy histology, pathological stage, preoperative hydronephrosis status, lymphovascular invasion status, surgical margin, tumor necrosis, pathological T and N stages, chemotherapy and radiotherapy status, surgical approach, pre-operative laboratory data, postoperative Clavien–Dindo classification, post-operative complications, and length of hospital stay were also recorded.

The primary endpoint of this study was to identify factors affecting the oncological outcomes in the CKD and ESRD groups with regard to overall survival (OS), cancer-specific survival (CSS), disease-free survival (DFS), and bladder recurrence-free survival (BRFS).

Differences between groups were compared using the two-sample Mann–Whitney U test for continuous variables, and Pearson’s chi-square test for categorical variables. Continuous variables were tested for normality with the Kolmogorov–Smirnov test. Kaplan–Meier curves were used to estimate the rates of prognostic outcomes, and the survival curves were compared using the stratified log-rank test. A Cox proportional hazard model was used to assess the effect of the surgical approach on the prognostic outcomes, alone and after adjusting for potential confounders. All statistical assessments were two-tailed and considered statistically significant at *p* < 0.05. Statistical analyses were carried out with SPSS version 26 (IBM Corp., Armonk, NY, USA).

## 3. Results

The demographic data, risk factors, and comorbidities of the patients are shown in Table 1. The male-to-female ratio was 1:1.4 (41:59) to 1:2.2 (31:69) in this study. Compared with the ESRD group, the CKD group was older (CKD vs. ESRD, 70.66 ± 9.84 vs. 64.96 ± 9.66 years, *p* < 0.001) and had a higher percentage of smokers (20.7% vs. 8.2%, *p* = 0.006), but lower rates of chemical exposure (3.3% vs. 9.4%, *p* = 0.008) and nephroureterectomy for UTUC (1.0% vs. 12.9%, *p* < 0.001). There were no significant differences in the listed comorbidities between the two groups.

Table 2 shows the demographic data, risk factors, and comorbidities of the UTUC patients. Compared with the ESRD group, a smaller proportion of the CKD group had an initial presentation of gross hematuria (CKD vs. ESRD, 59.8% vs. 80.0%, *p* < 0.001) and was less prone to incidental findings (6.4% vs. 14.1%, *p* = 0.011). In both groups, the tumors were more commonly located in the renal pelvis than in other locations. Tumor size was larger in the CKD group (*p* = 0.044). Tumor laterality was similar, and the histology was predominantly UC. The ESRD group had a higher percentage of high-grade tumors (CKD vs. ESRD, 76.0% vs. 91.8%, *p* = 0.014); however, the CKD group had a higher percentage of stage III and stage IV UTUC. Preoperative platelet, hemoglobin, and albumin levels were higher in the CKD group, but there were no significant differences in perioperative complications between the two groups. Of note, 129 patients in the CKD group progressed to ESRD after cancer treatment.

Factors affecting survival of the UTUC patients with CKD or ESRD in univariate analysis are shown in Supplementary Materials. There were no significant differences in OS, CSS, DFS, and BRFS between the two groups. The factors associated with OS were an age of >70 years, tumors located in the middle ureter, positive surgical margin, lymphovascular invasion, chemotherapy and radiotherapy for UTUC, high-grade tumors (stage III and IV), pathological stage III and IV, pathological pN1, and presentation of gross hematuria. The factors associated with CSS were tumors located in the middle ureter and bladder cuff, positive surgical margin, lymphovascular invasion, chemotherapy and radiotherapy for UTUC, high-grade tumors (stage III and IV), pathological stage III and IV, pathological pN1 and pN2, and presentation of gross hematuria. The factors associated with DFS were tumor size > 3 cm, middle/lower ureter tumors, bladder cuff tumors, lymphovascular invasion, positive surgical margin, chemotherapy for UTUC, radiotherapy for UTUC, high-grade tumors (stage III and IV), pathological stage pT3/pT4/pN1/pN2, and initial presentation of gross hematuria. The factors associated with BRFS were female sex, lower ureter tumors, history of cardiovascular events, arrhythmias, gout, smoking, and regular hair coloring.

**Figure 1:**
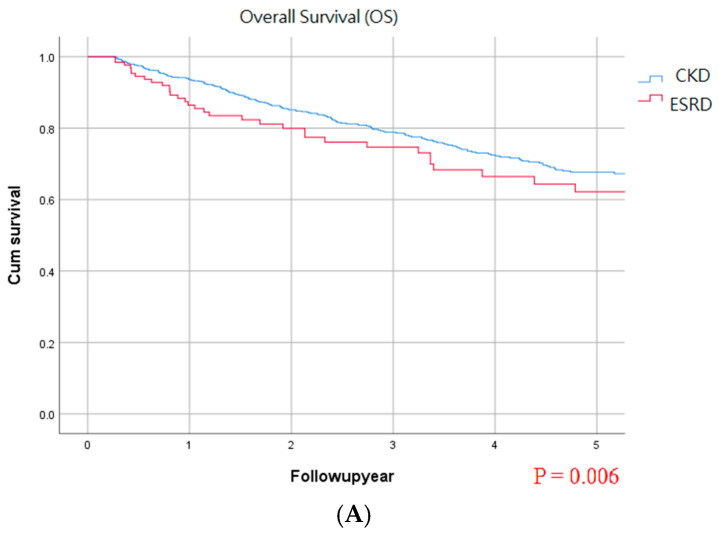

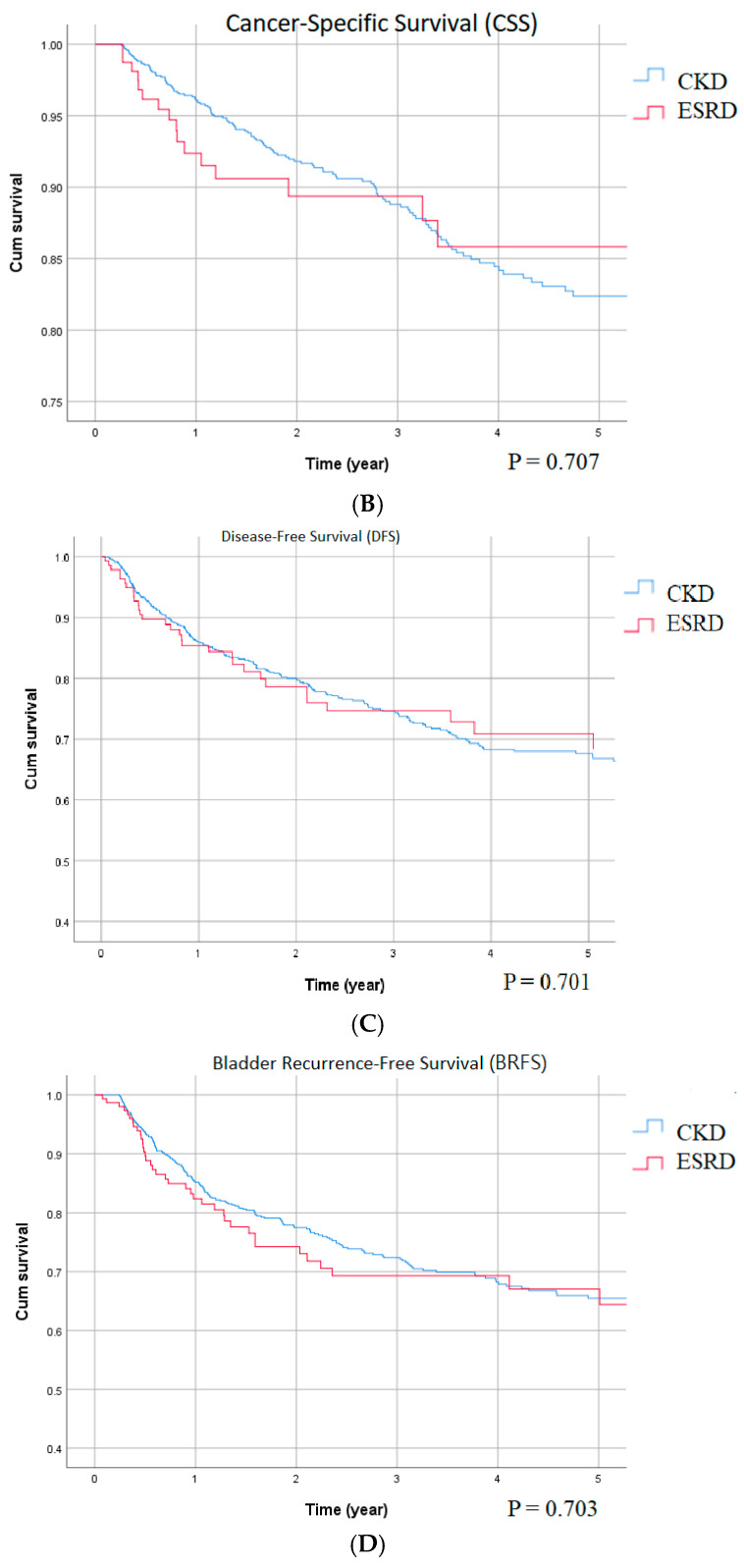
Five-year overall survival (**A**), cancer-specific survival (**B**), disease-free survival (**C**), and bladder recurrence-free survival (**D**) rates between the CKD and ESRD groups.

After multivariate analysis, ESRD, age > 70 years, positive surgical margin, and stage III and IV disease were associated with OS (Table 3). Middle ureter tumors, positive surgical margin, and stage III and IV disease were associated with CSS (Table 4). Middle and lower ureter tumors and stage III and IV disease were associated with poorer DFS (Table 5). Female sex, coronary artery disease, and arrhythmias were negatively related to BRFS, and lower ureter tumor was positively related to BRFS. CKD/ESRD status, history of gout, or smoking were not related to BRFS risks (Table 6). Figure 1 demonstrates the cumulative survival curve of the two groups in OS, CSS, DFS, and BRFS. Only the OS showed differences between the two groups.

## 4. Discussion

Although the prevalence and severity of UTUC and the incidence of CKD are different between Taiwan and Western countries, a comprehensive database of patients was lacking in Taiwan. Consequently, the Taiwan UTUC Collaboration Group was founded to better understand and promote further research in this UTUC endemic region. In this study, we focused on patients with CKD and ESRD, and found that the percentage of females was higher than males, with a male-to-female (M/F) ratio of 41:59 in the CKD group and 31:69 in the ESRD group. These findings are consistent with previous studies in Western countries, which reported a M/F ratio of 2:1 to 4:1 regardless of renal function [6,7], and a previous study in Taiwan, which reported a M/F ratio of 1:1.3 [8].

The management of UTUC in patients with CKD presents a complex diagnostic dilemma for the urologist, where the imperative for accurate oncological staging must be carefully weighed against the risk of iatrogenic renal injury. Patients with ESRD carry a profoundly elevated risk of UTUC, with standardized incidence ratios reported to be 7–18 times higher than the general population, a risk that is particularly pronounced in women [9]. This necessitates a high index of suspicion; however, the diagnostic pathway is fraught with challenges. The use of contrast-enhanced computed tomography urography (CTU) raises legitimate concern for contrast-induced nephropathy, potentially accelerating the decline of residual renal function in pre-dialysis patients or precipitating dialysis in others. Alternative imaging, including magnetic resonance urography, avoids nephrotoxic contrast but may provide inferior spatial resolution for small urothelial lesions. The definitive diagnostic modality, ureteroscopic biopsy, contains its own set of risks. Wang et al. emphasized a well-documented concern for tumor cell seeding or dissemination related to endoscopic procedure, which might potentially upstage disease and compromise oncologic outcomes [9]. Therefore, the urologist faces a critical decision: to pursue a definitive but risky tissue proof versus depending on less invasive, but possibly inconclusive, image studies. This challenge is compounded by the often non-specific presentation of UTUC in this population, whose symptoms may be falsely attributed to the underlying CKD or to the uremic condition, which may cause hematuria and alter immune surveillance [10]. As a matter of fact, creating a diagnostic algorithm for UTUC in CKD and ESRD patients requires a delicate and patient-directed approach that incorporates the degree of renal impairment, tumor suspicion, and the holistic goals of care, balancing the goal of cancer control with the paramount importance of renal function protection.

Univariate analysis found no significant differences in OS, CSS, DFS, and BRFS between the two groups, attributed to counterbalanced tumor characteristics: ESRD patients had higher-grade tumors, while CKD patients presented with more advanced Stage III/IV disease, confirming tumor grade/stage as core prognostic factors over renal function.

The results of this large-scale study showed that OS of the enrolled patients was associated with CKD/ESRD stage, age > 70 years, positive surgical margin, high-grade tumors in histology, and pathological stage III and IV. In addition, the ESRD patients had a 1.733-fold worse OS than their CKD counterparts. These findings are consistent with those of Hung et al. [5], who reported that the aggressiveness of UTUC was positively correlated with the severity of CKD, and that the patients with CKD stage 4/5 had more severe disease than those with CKD stage 1/2/3. This may be related to higher concentrations of carcinogens accumulating in the body as CKD worsens. Uremic toxins are immunosuppressive, which may aggravate the extent of cancer [11]. Progressive decline of renal function impairs the clearance of uremic toxins. Cohen et al. reported that the uremic toxins directly and indirectly compromise innate and adaptive immunity, creating a pro-inflammatory and pro-oxidant state [10]. The protein-bound solutes, such as indoxyl sulfate (IS) and p-cresyl sulfate (pCS), promote endothelial damage and leukocyte activation [10,12], stimulating interactions between leukocytes and the vascular wall, and promoting a chronic inflammatory environment which facilitates cellular damage and malignant transformation [12]. The sustained inflammation can lead to repeated epithelial injury and repair cycles in the urinary tract, which is a well-established risk factor for urothelial carcinogenesis.

In this study, CSS was associated with tumors located in the middle ureter, positive surgical margins, and pathological stage III and IV. In addition, DFS was associated with tumors located in the middle and lower ureter, and pathological stage III and IV. Different tumor locations in patients with UTUC may be related to different survival outcomes. Renal pelvic UC is surrounded by renal parenchyma, perirenal fat, and Gerota’s fascia, which may limit the spread of the tumor [13]. Ureteral UC is covered with only a thin layer of muscular and fatty tissue, so the resected specimens may have a higher chance of containing pathologically identifiable tumors or micro-metastasis [14]. Colin et al. reported a higher positive surgical margin rate in patients undergoing surgery for ureteral UC than in those undergoing renal pelvic UC surgery, which may explain the worse CSS in patients with ureteral UC [15].

Sheu et al. reported that tumor distribution affected bladder recurrence of multifocal UTUC treated with radical nephroureterectomy in Taiwan. They found that age > 67.6 years, male sex, history of gout, non-UC malignancy, history of bladder tumors, and synchronous renal pelvic and ureter tumors were related to BRFS [16]. However, they did not mention the renal function of the enrolled patients. In our cohort, we focused on UTUC patients with CKD and ESRD, and found that the CKD stage did not affect the BRFS. In addition, the effect of gout on BRFS was not prominent in our patients, even though it was associated with a three-fold higher risk of BRFS in Sheu et al.’s study. Coronary artery disease and arrhythmias showed some protective effects against BRFS; however, further studies are needed to clarify the correlation between heart disease and bladder recurrence of UTUC. Regardless, multidisciplinary oncological and cardiovascular follow-up for this population remains a cornerstone of contemporary urological cancer care.

Although this study provides data on specific groups of patients (e.g., CKD/ESRD) with UTUC from multiple centers in Taiwan, there are still some limitations. Firstly, due to the study design, we did not include a control group with normal renal function at the time of UTUC diagnosis. Secondly, this was a retrospective study, and the operations were performed by various surgeons with different methods, which may have caused bias. This can be overcome by Cox regression to specify independent risk factors. Thirdly, we did not conduct centralized pathological and radiological reviews. This can be overcome by applying a standard format based on the AJCC TNM staging system and NCCN guidelines.

In summary, this study validates core UTUC management principles and identifies population-specific considerations, guiding individualized, multidisciplinary care that balances oncological control and renal preservation for CKD/ESRD patients with UTUC.

## 5. Conclusions

In conclusion, in Taiwanese UTUC patients with CKD or ESRD in this study, OS was related to CKD stage and age over 70 years. Tumor location and high pathological stage were associated with OS, CSS, and DFS. BRFS was associated with female sex, tumor location, and cardiac diseases such as coronary artery disease and arrhythmias. Further studies should be conducted to elucidate the relationships between heart disease and UTUC survival, and also between the administration of ICI therapy and survival outcomes.

## Figures and Tables

**Table 1:** Demographic data of the patients with UTUC.

Variables	eGFR < 60 (N = 690)		CKD (N = 605)		ESRD (N = 85)		*p*-Value ^a^
	N	%	N	%	N	%	
Gender							
Men	274	(39.7)	248	(41.0)	26	(30.6)	0.066
Women	416	(60.3)	357	(59.0)	59	(69.4)	
Age. ^b^ Mean ± SD	69.92 ± 10.0		70.66 ± 9.84		64.96 ± 9.66		<0.001 ***
BMI. ^b^ Mean ± SD	24.51 ± 3.78		24.63 ± 3.71		23.80 ± 3.86		0.056
Risk factor							
Smoking	132	(19.1)	125	(20.7)	7	(8.2)	0.006 **
Chemical exposure	28	(4.1)	20	(3.3)	8	(9.4)	0.008 **
Herbal supplements	69	(10.0)	56	(9.3)	13	(15.3)	0.082
Arsenic water	50	(7.2)	42	(6.9)	8	(9.4)	0.411
Previous nephroureterectomy for UC	17	(2.5)	6	(1.0)	11	(12.9)	<0.001 ***
Regular hair coloring	34	(4.9)	29	(4.8)	5	(1.0)	0.664
Comorbidity							
CAD	74	(10.7)	64	(10.6)	10	(11.8)	0.741
Arrhythmias	27	(3.9)	24	(4.0)	3	(3.5)	0.846
HTN	388	(56.2)	341	(56.4)	47	(55.3)	0.852
DM	185	(26.8)	165	(27.3)	20	(23.5)	0.466
Gout	25	(3.6)	19	(3.1)	6	(7.1)	0.070
GI	80	(11.6)	66	(10.9)	14	(16.5)	0.134
Malignancy (No UTUC/bladder UC)	77	(11.2)	71	(11.7)	6	(7.1)	0.200

^a^ A chi-square test was used to calculate the difference between variables. ^b^ A Mann–Whitney U test was used to calculate the difference between continuous variables. ** *p* < 0.01, *** *p* < 0.001.

**Table 2:** Baseline demographic data, risk factors, and comorbidities of the UTUC patients.

Variables	eGFR < 60 (N = 690)		CKD (N = 605)		ESRD (N = 85)		*p*-Value ^a^
	N	%	N	%	N	%	
Initial presentation							
Gross hematuria	430	(62.3)	362	(59.8)	68	(80.0)	<0.001 ***
Flank pain	153	(22.2)	141	(23.3)	12	(14.1)	0.056
Fever	20	(2.9)	16	(2.6)	4	(4.7)	0.289
Hydronephrosis	43	(6.2)	41	(6.8)	2	(2.4)	0.114
Incidental finding	51	(7.4)	39	(6.4)	12	(14.1)	0.011 *
Microscopic hematuria	23	(3.3)	22	(3.6)	1	(1.2)	0.237
Weight loss	22	(3.2)	21	(3.5)	1	(1.2)	0.260
Tumor location							
Renal pelvis	440	(63.8)	382	(63.1)	58	(68.2)	0.360
Upper ureter	167	(24.2)	149	(24.6)	18	(21.2)	0.487
Middle ureter	99	(14.3)	83	(13.7)	16	(18.8)	0.209
Lower ureter	131	(19.0)	116	(19.2)	15	(17.6)	0.737
Bladder cuff	9	(1.3)	6	(1.0)	3	(3.5)	0.053
Tumor size							
Non-visible	5	(0.7)	4	(0.7)	1	(1.2)	0.044 *
<1 cm	43	(6.2)	31	(5.1)	12	(14.1)	
≥1 & <2 cm	125	(18.1)	105	(17.4)	20	(23.5)	
≥2 & <3 cm	124	(18.0)	106	(17.5)	18	(21.2)	
≥3 cm	309	(44.8)	275	(45.5)	34	(40.0)	
Affected kidney at diagnosis							
Left	324	(47.0)	281	(46.4)	43	(50.6)	0.156
Right	356	(51.6)	317	(52.4)	39	(45.9)	
Bilateral	7	(1.0)	5	(0.8)	2	(2.4)	
Graft kidney	2	(0.3)	1	(0.2)	1	(1.2)	
Cell type							
Urothelial	628	(91.0)	549	(90.7)	79	(92.9)	0.430
UC with variants	56	(8.1)	51	(8.4)	5	(5.9)	
Squamous	2	(0.3)	1	(0.2)	1	(1.2)	
Small cell	2	(0.3)	2	(0.3)	0	(0.0)	
Others	2	(0.3)	2	(0.3)	0	(0.0)	
Multiplicity							
Not available	9	(1.3)	7	(1.2)	2	(2.4)	0.132
No	473	(68.6)	422	(69.8)	51	(60.0)	
Yes	203	(29.4)	171	(28.3)	32	(37.6)	
Pre-op urine cytology							
Negative	140	(20.3)	120	(19.8)	20	(23.5)	0.940
Atypia	124	(18.0)	107	(17.7)	17	(20.0)	
Positive	209	(30.3)	182	(30.1)	27	(31.8)	
No cytology	124	(18.0)	105	(17.4)	19	(22.4)	
CIS							
No	354	(51.3)	312	(51.6)	42	(49.4)	0.904
Yes	81	(11.7)	71	(11.7)	10	(11.8)	
NUx histology							
Low grade	95	(13.8)	89	(14.7)	6	(7.1)	0.014 *
High grade	538	(78.0)	460	(76.0)	78	(91.8)	
G2	38	(5.5)	37	(6.1)	1	(1.2)	
Pathological stage							
Stage 0a/0is	111	(16.1)	93	(15.4)	18	(21.2)	0.014 *
Stage I	147	(21.3)	119	(19.7)	28	(32.9)	
Stage II	133	(19.3)	117	(19.3)	16	(18.8)	
Stage III	218	(31.6)	198	(32.7)	20	(23.5)	
Stage IV	62	(9.0)	59	(9.8)	3	(3.5)	
Preoperative hydronephrosis							
No	199	(28.8)	165	(27.3)	34	(40.0)	0.078
Yes	405	(58.7)	357	(59.0)	48	(56.5)	
Lymphovascular invasion							
No	514	(74.5)	447	(73.9)	67	(78.8)	0.472
Yes	148	(21.4)	132	(21.8)	16	(18.8)	
Surgical margin							
Free	575	(83.3)	492	(81.3)	83	(97.6)	0.067
Positive	20	(2.9)	20	(3.3)	0	0.0	
Tumor necrosis							
No	250	(36.2)	211	(34.9)	39	(45.9)	0.356
Yes	117	(17.0)	103	(17.0)	14	(16.5)	
Pathological stage T							
pTis	11	(1.6)	9	(1.5)	2	(2.4)	0.076
pTa	105	(15.2)	89	(14.7)	16	(18.8)	
pT0	148	(21.4)	120	(19.8)	28	(32.9)	
pT1	134	(19.4)	119	(19.7)	15	(17.6)	
pT2	236	(34.2)	217	(35.9)	19	(22.4)	
pT3	27	(3.9)	24	(4.0)	3	(3.5)	
pT4	2	(0.3)	2	(0.3)	0	(0.0)	
Pathological stage N							
pN0	119	(17.2)	102	(16.9)	17	(20.0)	0.484
pN1	14	(2.0)	14	(2.3)	0	(0.0)	
pN2	11	(1.6)	10	(1.7)	1	(1.2)	
pNx	517	(74.9)	452	(74.7)	65	(76.5)	
Chemotherapy for UTUC							
No	500	(72.5)	434	(71.7)	66	(77.6)	0.111
Yes	171	(24.8)	158	(26.1)	13	(15.3)	
Chemotherapy for UTUC							
Neo-adjuvant	15	(2.2)	13	(2.1)	2	(2.4)	0.648
Adjuvant	102	(14.8)	95	(15.7)	7	(8.2)	
Salvage (curative intent)	9	(1.3)	9	(1.5)	0	(0.0)	
Palliative (S/S relieve)	44	(6.4)	40	(6.6)	4	(4.7)	
Radiation therapy for UTUC							
No	602	(87.2)	527	(87.1)	75	(88.2)	0.097
Yes	70	(10.1)	66	(10.9)	4	(4.7)	
NxUx							
Open	195	(28.3)	175	(28.9)	20	(23.5)	0.038 *
Laparoscopic hand-assisted	294	(42.6)	258	(42.6)	36	(42.4)	
Robot-assisted	51	(7.4)	47	(7.8)	4	(4.7)	
Laparoscopy	137	(19.9)	114	(18.8)	23	(27.1)	
LESS	3	(0.4)	3	(0.5)	0	(0.0)	
NxUx							
Transperitoneal	274	(39.7)	234	(38.7)	40	(47.1)	0.019 *
Retroperitoneal	361	(52.3)	319	(52.7)	42	(49.4)	
Pre-OP Cr level (mg/dL). ^b^ Mean ± SD	2.66 ± 2.67		2.01 ± 1.66		7.25 ± 3.80		<0.001 **
Pre-OP Platelet (×10^3^/μL). ^b^ Mean ± SD	228.1 ± 73.8		233.6 ± 73.8		193.4 ± 60.29		<0.001 ***
Pre-OP Hgb. ^b^ Mean ± SD	11.31 ± 1.88		11.45 ± 1.87		10.36 ± 1.63		<0.001 ***
Pre-Albumin. ^b^ Mean ± SD	3.87 ± 0.63		3.91 ± 0.59		3.50 ± 0.79		0.032 *
Post-OP Cr level (mg/dL). ^b^ Mean ± SD	3.19 ± 2.73		2.80 ± 2.45		6.30 ± 2.90		<0.001 ***
Clavien–Dindo classification							
Grade I	68	(9.9)	60	(9.9)	8	(9.4)	0.214
Grade II	111	(16.1)	90	(14.9)	21	(24.7)	
Grade III	27	(3.9)	22	(3.6)	5	(5.9)	
Grade IV	26	(3.8)	23	(3.8)	3	(3.5)	
Grade V	1	(0.1)	1	(0.2)	0	(0.0)	
Post-OP complication							
ESRD	129	(18.7)	129	(21.3)	0	(0.0)	<0.001 ***
Ileus	15	(2.2)	11	(1.8)	4	(4.7)	0.070
Ventral hernia	5	(0.7)	4	(0.7)	1	(1.2)	0.566
Mortality							
No	321	(46.5)	281	(46.4)	40	(47.1)	0.314
UTUC-related	105	(15.2)	96	(15.9)	9	(10.6)	
non-UTUC-related	87	(12.6)	71	(11.7)	16	(18.8)	
Unknown	175	(25.4)	155	(25.6)	20	(23.5)	
Surgery-related	2	(0.3)	2	(0.3)	0	(0.0)	
Disease free							
No	197	(28.6)	180	(29.8)	17	(20.0)	0.260
Yes	443	(64.2)	384	(63.5)	59	(69.4)	
Hospital stay (days), ^c^ median	8.00		8.00		8.00		0.132
Follow-up (months), ^c^ median	34.92		35.25		34.72		0.946

^a^ A chi-square test was used to calculate the difference between variables. ^b^ A Mann–Whitney U test was used for differences in means. ^c^ A Wilcoxon rank-sum test was used for differences in medians. * *p* < 0.05, ** *p* < 0.01, *** *p* < 0.001.

**Table 3:** Multivariate analysis of the factors associated with overall survival.

Multivariate Analysis	OS	
	HR (95% CI)	*p*-Value
Group		
CKD	1	
ESRD	1.733 (1.074, 2.797)	0.024 *
Age		
<70	1	
≥70	1.775 (1.242, 2.538)	0.002 **
Middle ureter		
No	1	
Yes	1.303 (0.829, 2.047)	0.252
Lymphovascular invasion		
No	1	
Yes	0.891 (0.566, 1.404)	0.620
Surgical margin		
Free	1	
Positive	3.085 (1.623, 5.865)	0.001 **
NUx histology		
Low grade	1	
High grade	1.108 (0.626, 1.962)	0.725
Pathological stage		
Stage 0a/0is	1	
Stage I	1.151 (0.587, 2.257)	0.683
Stage II	1.387 (0.683, 2.815)	0.365
Stage III	2.237 (1.177, 4.252)	0.014 *
Stage IV	4.940 (2.221, 10.988)	<0.001 ***
Gross hematuria		
No	1	
Yes	0.778 (0.542, 1.115)	0.172

CI, confidence interval; HR, hazard ratio; OS, overall survival. * *p* < 0.05, ** *p* < 0.01, *** *p* < 0.001.

**Table 4:** Multivariate analysis of the factors associated with cancer-specific survival.

Multivariate Analysis	CSS	
	HR (95% CI)	*p*-Value
Group		
CKD	1	
ESRD	0.963 (0.446, 2.078)	0.923
Middle ureter		
No	1	
Yes	1.836 (1.026, 3.284)	0.041 *
Bladder cuff		
No	1	
Yes	0.650 (0.195, 2.171)	0.484
Lymphovascular invasion		
No	1	
Yes	1.070 (0.610, 1.876)	0.814
Surgical margin		
Free	1	
Positive	3.047 (1.388, 6.690)	0.005 **
NUx histology		
Low grade	1	
High grade	1.356 (0.510, 3.603)	0.542
Pathological stage		
Stage 0a/0is	1	
Stage I	0.824 (0.219, 3.100)	0.774
Stage II	1.620 (0.476, 5.513)	0.440
Stage III	4.272 (1.424, 12.814)	0.010 *
Stage IV	11.681 (3.448, 39.575)	<0.001 ***
Gross hematuria		
No	1	
Yes	0.771 (0.477, 1.248)	0.291

CI, confidence interval; HR, hazard ratio; CSS, cancer-specific survival. * *p* < 0.05, ** *p* < 0.01, *** *p* < 0.001.

**Table 5:** Multivariate analysis of the factors associated with disease-free survival.

Multivariate Analysis	DFS	
	HR (95% CI)	*p*-Value
Group		
CKD	1	
ESRD	0.856 (0.489, 1.497)	0.585
Tumor size		
<1 cm	1	
≥1 & <2 cm	1.208 (0.394, 3.698)	0.741
≥2 & <3 cm	1.211 (0.398, 3.686)	0.736
≥3 cm	1.694 (0.589, 4.878)	0.328
Middle ureter		
No	1	
Yes	2.100 (1.378, 3.200)	0.001 **
Lower ureter		
No	1	
Yes	1.714 (1.139, 2.580)	0.010 *
Bladder cuff		
No	1	
Yes	1.472 (0.508, 4.269)	0.476
Lymphovascular invasion		
No	1	
Yes	1.297 (0.846, 1.990)	0.233
Surgical margin		
Free	1	
Positive	1.703 (0.804, 3.609)	0.165
NUx histology		
Low grade	1	
High grade	1.453 (0.745, 2.835)	0.273
Pathological stage		
Stage 0a/0is	1	
Stage I	0.759 (0.341, 1.692)	0.500
Stage II	1.135 (0.521, 2.469)	0.750
Stage III	2.146 (1.041, 4.424)	0.039 *
Stage IV	5.084 (2.170, 11.912)	<0.001 ***
Gross hematuria		
No	1	
Yes	1.072 (0.735, 1.566)	0.717

CI, confidence interval; HR, hazard ratio; DFS, disease-free survival. * *p* < 0.05, ** *p* < 0.01, *** *p* < 0.001.

**Table 6:** Multivariate analysis of the factors associated with bladder recurrence-free survival.

Multivariate Analysis	BRFS	
	HR (95% CI)	*p*-Value
Group		
CKD	1	
ESRD	1.140 (0.733, 1.775)	0.561
Sex		
Male	1	
Female	0.678 (0.481, 0.956)	0.027 *
Lower ureter		
No	1	
Yes	1.490 (1.046, 2.124)	0.027 *
CAD		
No	1	
Yes	0.521 (0.274, 0.991)	0.047 *
Arrythmia		
No	1	
Yes	0.132 (0.019, 0.948)	0.044 *
Gout		
No	1	
Yes	1.435 (0.768, 2.684)	0.258
Smoking		
No	1	
Yes	1.313 (0.878, 1.963)	0.184

CI, confidence interval; HR, hazard ratio; BRFS, bladder recurrence-free survival. * *p* < 0.05.

## Data Availability

The original contributions presented in this study are included in the article and Supplementary Material. Further inquiries can be directed to the corresponding author.

## Supplementary Materials

The following supporting information can be downloaded at: https://www.mdpi.com/article/10.3390/biomedicines14030554/s1, Table S1: Univariate analysis of the factors associated with survival.
