# Supplementary material for: Factors Affecting Oncological Outcomes in Upper Tract Urothelial Carcinoma Patients with Chronic Kidney Disease and End-Stage Renal Disease
Source: Biomedicines. 2026 Feb 28;14(3):554. doi: 10.3390/biomedicines14030554 (PMC13023803; doi:10.3390/biomedicines14030554)
Supplement: Supplementary file 1 [file biomedicines-14-00554-s001.zip › biomedicines-4116131-supplementary.pdf]

Table S1. Univariate analysis of the factors associated with survival

Univariate analysis	OS		CSS		DFS		BRFS	
	HR (95% CI)	p-value	HR(95% CI)	p-value	HR(95% CI)	p-value	HR(95% CI)	p-value
Group								
CKD	1		1		1		1	
ESRD	1.083 (0.712, 1.650)	0.709	0.669 (0.338, 1.324)	0.249	0.676 (0.411, 1.112)	0.123	1.094 (0.705, 1.696)	0.689
Sex								
Male	1		1		1		1	
Female	0.911 (0.685, 1.212)	0.521	0.810 (0.553, 1.186)	0.280	1.000 (0.752, 1.328)	0.998	0.585 (0.435, 0.787)	<0.001**
Age								
<70	1		1		1		1	
>=70	1.340 (1.008, 1.783)	0.044*	1.270 (0.866, 1.862)	0.221	1.196 (0.903, 1.583)	0.213	1.027 (0.764, 1.381)	0.861
BMI								
<24	1		1		1		1	
>=24	1.032 (0.703, 1.517)	0.871	1.298 (0.759, 2.218)	0.341	1.004 (0.688, 1.466)	0.982	0.968 (0.664, 1.413)	0.868
CIS								
No	1		1		1		1	
Yes	0.777 (0.478, 1.265)	0.311	0.671 (0.333, 1.352)	0.264	1.079 (0.698, 1.667)	0.732	1.209 (0.739, 1.978)	0.450
Tumor size								
<1cm	1		1		1		1	
≥1 & < 2 cm	1.125 (0.509, 2.486)	0.771	0.882 (0.276, 2.813)	0.832	1.074 (0.457, 2.527)	0.870	0.849 (0.467, 1.541)	0.590
≥2 & < 3 cm	1.476 (0.676, 3.222)	0.328	1.090 (0.347, 3.424)	0.883	1.443 (0.626, 3.324)	0.389	0.763 (0.414, 1.407)	0.386

≥ 3cm	1.778 (0.860, 3.676) 0.120	2.280 (0.826, 6.295) 0.112	2.372 (1.102, 5.106) 0.027*	0.612 (0.349, 1.075) 0.087
Renal pelvis				
No	1	1	1	1
Yes	0.886 (0.663, 1.183) 0.412	0.815 (0.554, 1.199) 0.299	0.782 (0.590, 1.037) 0.088	0.831 (0.614, 1.126) 0.232

(Continued on the next page)

Table S1. Univariate analysis of the factors associated with survival (con.)

Univariate analysis	OS		CSS		DFS		BRFS	
	HR (95% CI)	p-value	HR(95% CI)	p-value	HR(95% CI)	p-value	HR(95% CI)	p-value
Upper ureter								
No	1		1		1		1	
Yes	0.883 (0.629, 1.240)	0.474	0.752 (0.467, 1.212)	0.242	0.818 (0.580, 1.154)	0.253	1.101 (0.782, 1.550)	0.581
Middle ureter								
No	1		1		1		1	
Yes	1.497 (1.033, 2.170)	0.033*	1.949 (1.237, 3.071)	0.004**	1.857 (1.316, 2.621)	<0.001**	1.167 (0.764, 1.783)	0.475
Lower ureter								
No	1		1		1		1	
Yes	1.249 (0.884, 1.763)	0.207	1.399 (0.894, 2.189)	0.142	1.560 (1.132, 2.150)	0.007**	1.544 (1.088, 2.192)	0.015*
Bladder cuff								
No	1		1		1		1	
Yes	1.857 (0.763, 4.520)	0.173	3.610 (1.468, 8.875)	0.005**	3.389 (1.503, 7.642)	0.003**	1.935 (0.717, 5.223)	0.192
Multiplicity								
No	1		1		1		1	
Yes	1.239 (0.916, 1.675)	0.165	1.301 (0.869, 1.947)	0.201	1.435 (1.071, 1.924)	0.016	1.262 (0.918, 1.736)	0.152

Preoperative hydronephrosis								
No	1		1		1		1	
Yes	1.437 (0.998, 2.069)	0.051	1.487 (0.906, 2.440)	0.116	1.390 (0.975, 1.982)	0.069	1.301 (0.917, 1.845)	0.140
Lymphovascular invasion								
No	1		1		1		1	
Yes	1.556 (1.116, 2.171)	0.009**	2.528 (1.689, 3.784)	<0.001**	2.359 (1.752, 3.176)	<0.001**	0.992 (0.670, 1.471)	0.970

(Continued on the next page)

Table S1. Univariate analysis of the factors associated with survival (con.)

Univariate analysis	OS		CSS		DFS		BRFS	
	HR (95% CI)	p-value	HR(95% CI)	p-value	HR(95% CI)	p-value	HR(95% CI)	p-value
Surgical margin								
free	1		1		1		1	
positive	5.027 (2.830, 8.931)	<0.001**	7.094 (3.735, 13.474)	<0.001**	4.176 (2.359, 7.395)	<0.001**	0.949 (0.302, 2.982)	0.929
Tumor Necrosis								
No	1		1		1		1	
Yes	1.396 (0.929, 2.098)	0.108	1.718 (0.964, 3.061)	0.066	1.403 (0.924, 2.130)	0.112	1.240 (0.794, 1.935)	0.344
Chemotherapy for UTUC								
No	1		1		1		1	
Yes	3.263 (2.429, 4.384)	<0.001**	6.809 (4.573, 10.137)	<0.001**	4.975 (3.730, 6.635)	<0.001**	0.946 (0.656, 1.366)	0.769
Radiation therapy for UTUC								
No	1		1		1		1	

Yes	3.597 (2.536, 5.103)	<0.001**	5.908 (3.945, 8.848)	<0.001**	5.603 (4.073, 7.708)	<0.001**	1.037 (0.610, 1.764)	0.893

(Continued on the next page)

Table S1. Univariate analysis of the factors associated with survival (con.)

Univariate analysis	OS		CSS		DFS		BRFS	
	HR (95% CI)	p-value	HR(95% CI)	p-value	HR(95% CI)	p-value	HR(95% CI)	p-value
NUx histology								
low grade	1		1		1		1	
high grade	1.906 (1.182, 3.072)	0.008**	3.531 (1.546, 8.062)	0.003**	2.835 (1.612, 4.987)	<0.001**	0.657 (0.452, 0.954)	0.027
Pathological stage								
stage 0a/0is	1		1		1		1	
stage I	1.152 (0.657, 2.020)	0.622	0.818 (0.264, 2.538)	0.729	0.888 (0.462, 1.709)	0.723	0.855 (0.553, 1.323)	0.482
stage II	1.569 (0.901, 2.734)	0.112	2.179 (0.827, 5.737)	0.115	2.023 (1.129, 3.624)	0.018*	0.798 (0.502, 1.270)	0.342
stage III	2.755 (1.693, 4.485)	<0.001**	6.169 (2.641, 14.411)	<0.001**	3.441 (2.040, 5.806)	<0.001**	0.759 (0.493, 1.167)	0.209
stage IV	5.227 (3.005, 9.091)	<0.001**	15.372 (6.364, 37.132)	<0.001**	7.983 (4.521, 14.097)	<0.001**	0.499 (0.233, 1.070)	0.074
Pathological stage T								
pTis/pTa/pT0	1		1		1		1	

pT1	1.210 (0.700, 2.089)	0.495	0.857 (0.276, 2.657)	0.789	0.880 (0.462, 1.677)	0.698	0.931 (0.604, 1.437)	0.748
pT2	1.593 (0.921, 2.754)	0.096	2.505 (0.962, 6.525)	0.060	2.150 (1.220, 3.789)	0.008**	0.910 (0.576, 1.437)	0.685
pT3	3.181 (1.984, 5.101)	<0.001**	7.874 (3.395, 18.259)	<0.001**	3.847 (2.320, 6.379)	<0.001**	0.835 (0.546, 1.277)	0.405
pT4	5.787 (2.952, 11.343)	<0.001**	18.479 (7.002, 48.770)	<0.001**	11.288 (5.984, 21.292)	<0.001**	0.159 (0.022, 1.162)	0.070
Pathological stage N								
pN0	1		1		1		1	
pN1	4.166 (1.864, 9.310)	0.001*	7.785 (3.167, 19.135)	<0.001**	4.361 (2.116, 8.990)	<0.001**	1.156 (0.409, 3.267)	0.785
pN2	2.014 (0.771, 5.258)	0.153	4.315 (1.519, 12.258)	0.006**	2.774 (1.214, 6.339)	0.015*	0.510 (0.122, 2.126)	0.355

(Continued on the next page)

Table S1. Univariate analysis of the factors associated with survival (con.)

Univariate analysis	OS		CSS		DFS		BRFS	
	HR (95% CI)	p-value	HR(95% CI)	p-value	HR(95% CI)	p-value	HR(95% CI)	p-value
CAD								
No	1		1		1		1	
Yes	1.456 (0.956, 2.219)	0.080	1.287 (0.720, 2.299)	0.395	1.076 (0.684, 1.692)	0.752	0.507 (0.268, 0.960)	0.037*
Arrhythmia								
No	1		1		1		1	
Yes	0.852 (0.401, 1.813)	0.678	0.432 (0.107, 1.751)	0.240	0.363 (0.116, 1.134)	0.081	0.116 (0.016, 0.827)	0.032**
HTN								
No	1		1		1		1	
Yes	1.092 (0.820, 1.454)	0.546	0.819 (0.560, 1.197)	0.302	0.872 (0.659, 1.155)	0.340	1.078 (0.798, 1.456)	0.626
DM								
No	1		1		1		1	
Yes	1.111 (0.811, 1.522)	0.512	0.810 (0.514, 1.277)	0.365	0.914 (0.662, 1.263)	0.587	0.995 (0.709, 1.398)	0.979
Gout								
No	1		1		1		1	
Yes	0.497 (0.185, 1.338)	0.166	0.223 (0.031, 1.598)	0.135	0.482 (0.179, 1.296)	0.148	1.969 (1.069, 3.628)	0.030*
GI								
No	1		1		1		1	
Yes	0.894 (0.574, 1.394)	0.622	0.643 (0.325, 1.273)	0.205	0.876 (0.557, 1.378)	0.566	1.149 (0.747, 1.769)	0.527
Malignancy (not UTUC/ bladder UC)								
No	1		1		1		1	
Yes	1.375 (0.902, 2.095)	0.139	1.107 (0.607, 2.020)	0.741	0.878 (0.547, 1.411)	0.592	1.046 (0.642, 1.706)	0.856

(Continued on the next page)

Table S1. Univariate analysis of the factors associated with survival (con.)

Univariate analysis	OS		CSS		DFS		BRFS	
	HR (95% CI)	p-value	HR(95% CI)	p-value	HR(95% CI)	p-value	HR(95% CI)	p-value
Smoking								
No	1		1		1		1	
Yes	0.925 (0.638, 1.339)	0.678	1.305 (0.834, 2.042)	0.244	1.108 (0.785, 1.562)	0.560	1.642 (1.166, 2.310)	0.004**
Chemical exposure								
No	1		1		1		1	
Yes	0.711 (0.292, 1.729)	0.452	0.719 (0.228, 2.267)	0.573	0.800 (0.355, 1.803)	0.590	0.873 (0.387, 1.973)	0.745
Herbal supplements								
No	1		1		1		1	
Yes	0.594 (0.345, 1.024)	0.061	0.817 (0.426, 1.568)	0.543	0.947 (0.596, 1.504)	0.817	0.788 (0.471, 1.319)	0.365
Arsenic water								
No	1		1		1		1	
Yes	1.077 (0.645, 1.800)	0.776	1.221 (0.636, 2.343)	0.549	1.116 (0.669, 1.861)	0.675	0.833 (0.463, 1.499)	0.542
Previous Nephroureterectomy for UC								
No	1		1		1		1	
Yes	1.309 (0.538, 3.185)	0.553	0.872 (0.215, 3.535)	0.848	0.959 (0.356, 2.582)	0.934	1.216 (0.499, 2.961)	0.666
Regular Hair coloring								
No	1		1		1		1	
Yes	0.656 (0.308, 1.397)	0.274	0.661 (0.243, 1.796)	0.417	0.872 (0.447, 1.702)	0.688	0.279 (0.089, 0.875)	0.029*

(Continued on the next page)

Table S1. Univariate analysis of the factors associated with survival (con.)

Univariate analysis	OS		CSS		DFS		BRFS	
	HR (95% CI)	p-value	HR(95% CI)	p-value	HR(95% CI)	p-value	HR(95% CI)	p-value
Gross hematuria								
No	1		1		1		1	
Yes	0.534 (0.403, 0.708)	<0.001**	0.484 (0.331, 0.708)	<0.001**	0.638 (0.482, 0.843)	0.002**	1.142 (0.837, 1.559)	0.403
Flank pain								
No	1		1		1		1	
Yes	1.040 (0.741, 1.460)	0.820	1.049 (0.666, 1.653)	0.836	1.167 (0.841, 1.619)	0.356	0.716 (0.481, 1.067)	0.101
Fever								
No	1		1		1		1	
Yes	0.964 (0.358, 2.597)	0.942	1.279 (0.405, 4.037)	0.674	1.208 (0.497, 2.939)	0.676	0.953 (0.351, 2.585)	0.924
Hydronephrosis								
No	1		1		1		1	
Yes	1.024 (0.557, 1.884)	0.938	0.815 (0.332, 2.001)	0.655	0.669 (0.329, 1.357)	0.265	0.959 (0.506, 1.816)	0.898
Incidental finding								
No	1		1		1		1	
Yes	1.057 (0.613, 1.822)	0.841	1.208 (0.610, 2.391)	0.588	0.919 (0.524, 1.613)	0.769	1.217 (0.717, 2.066)	0.467
Microscopic hematuria								
No	1		1		1		1	
Yes	0.615 (0.273, 1.389)	0.243	0.611 (0.194, 1.928)	0.401	0.454 (0.168, 1.222)	0.118	0.992 (0.487, 2.019)	0.982

Weight loss				
No	1	1	1	1
Yes	0.309 (0.077, 1.246) 0.099	0.553 (0.136, 2.239) 0.406	1.276 (0.629, 2.589) 0.500	1.675 (0.823, 3.410) 0.155

CI, confidence interval; HR, hazard ratio; OS, overall survival; CSS, cancer-specific survival; DFS, disease-free survival; BRFS, Bladder Recurrence-free survival

p * < 0.05, ** p < 0.
